# Hypokalemic Periodic Paralysis Exacerbated by Carbohydrate Load: A Case Report

**DOI:** 10.7759/cureus.28851

**Published:** 2022-09-06

**Authors:** Ryan Blanton, Safi Afzal

**Affiliations:** 1 Emergency Medicine, Campbell University School of Osteopathic Medicine, Lillington, USA; 2 Internal Medicine, Cape Fear Valley Medical Center, Fayetteville, USA

**Keywords:** hypokalemia, na-k atpase, magnesium, diet, insulin, potassium, carbohydrate load, hypokalemic periodic paralysis

## Abstract

A 35-year-old male presented with weakness in all four extremities rendering him unable to ambulate. The patient stated the symptoms began after consuming an unknown, large amount of Oreo cookies; thus, a high carbohydrate load likely caused him to exceed the recommended dietary allowance (RDA) of 225-325 grams of carbohydrates per day, depending on one’s daily caloric intake. Lab workup revealed a potassium level of 2.1 mmol/L. Upon potassium replacement, the patient's symptoms improved to baseline, and he was discharged home with follow-up instructions that included a referral for genetic testing. Hypokalemic periodic paralysis (HPP) is a rare condition that, despite having a relatively simple solution for treatment, can lead to an extensive and expensive workup if not considered early on the list of differential diagnoses. Herein, we will discuss the pathophysiology, clinical signs/symptoms, and management of HPP.

## Introduction

Rare disorders such as hypokalemic periodic paralysis (HPP) are not always at the forefront of a clinician's mind when formulating a mental list of differential diagnoses, but they are incredibly important to consider when diagnosing patients, as they can protect patients from increased healthcare costs, prolonged hospital stays, and diagnostic procedural-associated risk. HPP is a rare neuromuscular disorder associated with ion channel disturbances and characterized by painless muscle weakness that may be potentially exacerbated by a variety of factors such as diet and/or exercise. To understand how HPP occurs, one must first analyze the effects of insulin on electrolyte regulation. An increased glucose load stimulates insulin release from pancreatic beta cells. Glucose transporter type 4 (GLUT4) transporters are activated in skeletal muscle, allowing glucose to enter the cell. Insulin also activates Na-H antiporters on the cell membrane, enabling sodium to enter cells, which in turn activates Na-K ATPase [1]. When Na-K ATPase is activated, an influx of potassium occurs via active transport in the setting of high-energy metabolic states. Periodic paralysis has been known to be associated with other conditions aside from potassium abnormalities such as thyrotoxicosis [2]. These disorders are typically diagnosed based on clinical presentation before being confirmed with genetic testing. Of note, the HPP gene was localized to chromosome 1q31-q32 near a dihydropyridine receptor gene critical for excitation-contraction coupling [3]. This finding highlights the possibility of a genetic component in this patient’s clinical presentation and symptomatology. This case specifically focuses on HPP exacerbated by high carbohydrate loads.

## Case presentation

**Figure 1:**
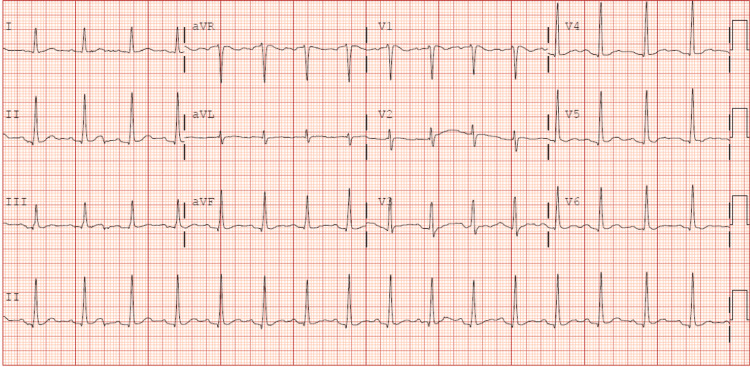
Patient's ECG in the setting of hypokalemic periodic paralysis

A 35-year-old male presented to the emergency department via emergency medical services for weakness in all four extremities and core muscles and an inability to ambulate that had begun the previous night. When questioned about the events leading up to his current presentation, he denied any heavy physical exertion and stated he was playing video games and had eaten an unknown, large amount of Oreo cookies the previous night before going straight to bed. Upon chart review, the patient had a similar presentation in 2010 that sparked an extensive workup with multiple physicians involved in his care and two lumbar punctures at the time. Apart from hypertension, other vital signs were within normal limits and a body mass index of 37.64 kg/m^2^ was calculated. He was alert and oriented to person, place, and time. On motor testing, he displayed 1/5 strength in upper extremities, and 0/5 in lower extremities bilaterally. Electrocardiography (ECG) revealed sinus tachycardia with a prolonged PR interval measuring 212 ms (Figure 1). Lab studies in the emergency department were significant for a potassium level of 2.1 mmol/L, total creatine kinase (CK) of 717 U/L, and thyroid-stimulating hormone (TSH) of 1.160 uIU/mL. The patient was given 40 mEq of potassium orally and an additional 40 mEq via IV while in the emergency department and was admitted to internal medicine service for further management. Repeat blood work revealed a potassium level of 4.7 mmol/L. Subsequently, the following morning, the patient endorsed the resolution of all symptoms that had led to his hospital admission. A repeat basic metabolic panel was drawn showing potassium of 3.5 mmol/L. He was then given an additional 40 mEq of oral potassium with a recommendation for primary care follow-up and continued oral potassium supplementation upon discharge.

## Discussion

The mechanism by which HPP occurs in the setting of a high carbohydrate load may be understood by analyzing the physiology and effect of an increased glucose load on insulin. Carbohydrates may be either simple or complex carbohydrates. Simple carbohydrates are composed of shorter chains and thus are metabolized faster than complex carbs [4]. This leads to sudden increases in serum glucose and insulin release. According to the 2020-2025 Dietary Guidelines for Americans, one should receive between 45% and 65% of the daily caloric intake from carbohydrates [5]. In a 2,000 calories per day diet, this comes out to be between 225 and 325 grams of carbohydrates per day. According to Oreo’s website, in one Oreo cookie, there are roughly 8.3 grams of carbohydrates [6].

Simple carbohydrates stimulate rapid insulin release, which activates a variety of downstream effects on electrolyte transport. Na-K ATPase is a primary mechanism by which a high carbohydrate load results in increased potassium uptake in cells, thus decreasing serum potassium levels. The consequences of decreased serum potassium levels can range from generalized weakness and fatigue to cardiac arrhythmias with severe neuromuscular weakness [7]. Hypokalemic changes can manifest electrocardiogram changes, beginning with decreasing T-wave amplitude and progressing to ST-segment depressions, T-wave inversions, and the characteristic U-wave [8]. If hypokalemia is left untreated, it can lead to potentially fatal tachyarrhythmias such as ventricular tachycardia, ventricular fibrillation, and atrioventricular block [8].

In cases of hypokalemia, the underlying cause should be identified. Genetic testing should be performed when other diagnoses are determined to be less likely through a negative workup. In cases where genetic testing for this disorder is negative, supervised in-patient provocative testing and electromyography may prove beneficial in finding the underlying cause [3]. Of note, metabolic acidosis/alkalosis can be a particularly important finding after one case series showed these derangements occurring in patients with secondary hypokalemia, but not in HPP [2]. Treatment for HPP consists of incremental potassium repletion without IV dextrose, as this can produce an exaggerated insulin response to a carbohydrate load [9]. Oral potassium is preferred over the intravenous route to avoid causing hyperkalemia [7]. In the event of severe hypokalemia causing clinical symptoms such as paralysis, both IV and oral potassium supplementation may be beneficial. These patients should be monitored for arrhythmia with an infusion rate no greater than 20 mmol per hour [7]. Checking the magnesium level is also warranted, as magnesium plays a role in the cellular uptake of potassium through the activation of Na-K ATPase pumps [7]. If magnesium is also depleted, hypokalemia may be refractory to supplementation so the hypomagnesemia will need to be replenished in conjunction with potassium. Diuretics and carbonic anhydrase inhibitors may also be used in the treatment of HPP [10]. Patients suffering from HPP can take preventative measures that may reduce future episodes by following recommended dietary guidelines, daily potassium supplementation, and by avoiding triggers [10] such as vigorous exercise and/or carbohydrate loads exceeding the recommended dietary allowance (RDA).

## Conclusions

Generalized weakness is a common complaint with a broad differential. HPP should be on a clinician’s list of differential diagnoses when a patient presents with weakness, especially when electrolyte abnormalities are discovered during lab workup. Early consideration of HPP can potentially lead to earlier diagnosis and treatment, which can improve patient care. In any case, if a diagnosis of periodic paralysis is suspected, then, in addition to potassium levels testing, labs should include thyroid function, ECG, and arterial blood gas (ABG) to rule out other diagnoses such as hyperthyroidism, Andersen syndrome, and metabolic acidosis/alkalosis and assist in the faster diagnosis of hypokalemia.
